# Sustainable production and preparative purification of thermostable alkaline α-amylase by *Bacillus simplex* (ON754233) employing natural deep eutectic solvent-based extractive fermentation

**DOI:** 10.1038/s41598-024-51168-7

**Published:** 2024-01-04

**Authors:** Ramya Muniasamy, Senthilkumar Rathnasaamy

**Affiliations:** Green Separation Engineering Laboratory, School of Chemical and Biotechnology, SASTRA Deemed to Be University, Thanjavur, Tamilnadu India

**Keywords:** Chemical engineering, Proteins

## Abstract

Using PEG-based deep eutectic solvents (PDES), the current study proposes extractive fermentation as a sustainable process integration for the production and purification of α-amylase from *Bacillus simplex* (ON754233). Glucose: PEG 400 outperformed five PDES in terms of tie lie length (58) and slope value (1.23) against sodium sulphatt. Apple cider pomace was used as a low-cost, sustainable carbon source to produce-amylase, with a maximum enzyme production of 2200.13 U/mL. PDES concentration (20% w/v), salt (12.75 w/v), and apple waste (2.75 g/mL) were all optimized using response surface methodology. When scaled upto 3 L benchtop bioreactor, extractive fermentation was proved to be better technology with maximum recovery of 92.4% with highest partition coefficient (3.59). The partially purified enzyme was further purified using a Sephadex G 100 followed by DEAE-Sephadex anion exchange chromatography with a purity fold of 33. The enzyme was found to be thermostable at the temperature (60 °C), remains alkaline (pH 8), and the activity was stimulated in the presence of Mg^2+^ ions. With SDS PAGE electrophoresis, the molecular weight was found to be around 140 kDa. Finally, the enzyme kinetics parameters were evaluated with observed K_m_ (0.00396 mM) and V_max_ (37.87 U/mL). Thus scaling up extractive fermentation entails increasing production capacity with improved extraction efficiency using green solvents.

## Introduction

α-amylase (E.C.3.2.1.1) is an industrially significant enzyme that catalyzes the hydrolysis of complex carbohydrates such as starch and glycogen, converting them into simpler sugars like maltose and glucose. It is produced by several plants during their growth phase of seed germination where conversion of starch into simple sugars is essential for survival^1^. Further, this workhorse enzyme has found numerous applications in various industries such as food production, brewing, biofuel production, and textile processing making it a key participant of commercial importance^2,3^ With the applications of this enzyme rapidly increasing over recent days, experts delve into the possibility of harnessing production of thermostable microbial amylase by *Bacillus sp.* to meet the growing needs^4–6^. Over recent decades, valorization of agro-forestry and food industry wastes like wood chips, fruit, and vegetable peels have been widely incorporated to emphasize the sustainable production of commercial enzymes^7,8^. Additionally, using these waste materials for commercial enzyme production seems more appealing due to its reasonable capital investment, simplicity of operation, low level of catabolite repression, and improved product recovery^9^. However, the use of these solid wastes has numerous disadvantages like the nature of the solid substrate and regulation of moisture content in the substrate^10–14^. The modification of moisture level in the chosen substrate influences the economics of the process^15^. This requires an alternate technology to ascertain the efficacy of the current world scenario, which accentuates a cost-effective sustainable approach for the production of α-amylases.

Extractive fermentation (EXFEM) is a pioneering technological integration of fermentation and extraction as a single step to facilitate the direct recovery of the partially pure enzyme from the production media. EXFEM employs aqueous two-phase partitioning (ATPS) that enables the partitioning of target molecules into one phase while infiltrating the microbial biomass and unutilized substrates into the alternate phase. This in-situ purification method overcomes the complexity such as low product yield through end-product inhibition and extraction of labile products that degrade before the completion of fermentation duration^16,17^. Earlier investigations have employed polyethylene glycol (PEG)—potassium phosphate biphasic systems for recovery of microbial amylase. The enzyme is observed to be infiltrated in the salt-rich phase due to increase in excluded volume resulting in a purity fold of 5.4 yielding 45.2%. When the PEG chain length increases the hydrophobicity of the PEG rich phase increased with the least space available for the protein^18^. When using PEG based aqueous two phase system, improvement in the energy cost reduction was observed during saccharification of starch (11 h) compared to conventional hydrolysis (18 h)^19^. Moreover the partitioning of amylase was found to be thermodynamically favorable due to the cations present in the salt rather than the molar mass of PEG^20^. Furthermore, PEG production required more energy and the accumulation of polymer wastes have an environmental impact. Consequently, the PEG extraction system appears to be time-consuming due to limitations in selectivity, polymer settlements, and solvent recovery considered critical in EXFEM to maintain the economic viability of the process. This issue could be circumvented by the introduction of deep eutectic solvents (DES) as alternates in the EXFEM process^21,22^.

Moreover, the economics of the greener solvents may successfully encourage the development of cost-effective bioprocess firms as a thermodynamically favorable process integration^23^. In regards, DES has several advantages such as simple preparation, low cost, and direct utilization in the process without prior purification have aroused interest in the extraction of target molecules^23,24^. Some of the widely reported extractive fermentation products include fibrinolytic protease^25^, lipase^26^, Pullans^27^, Penicillin^28^, alcohols^29^, and a range of carboxylic acids^30^. Although DES have overcome many drawback of conventional solvent system such as low toxicity, non-biodegradability, only limited investigations were observed on DES assisted EXFEM. There is currently a dearth of information regarding the EXFEM of amylase using DES despite numerous research that has addressed the conventional ATPS partitioning using the PEG-salt system. This has led to more investigation into the extractive fermentation and purification of alpha-amylase using green solvents.

**Figure 1:**
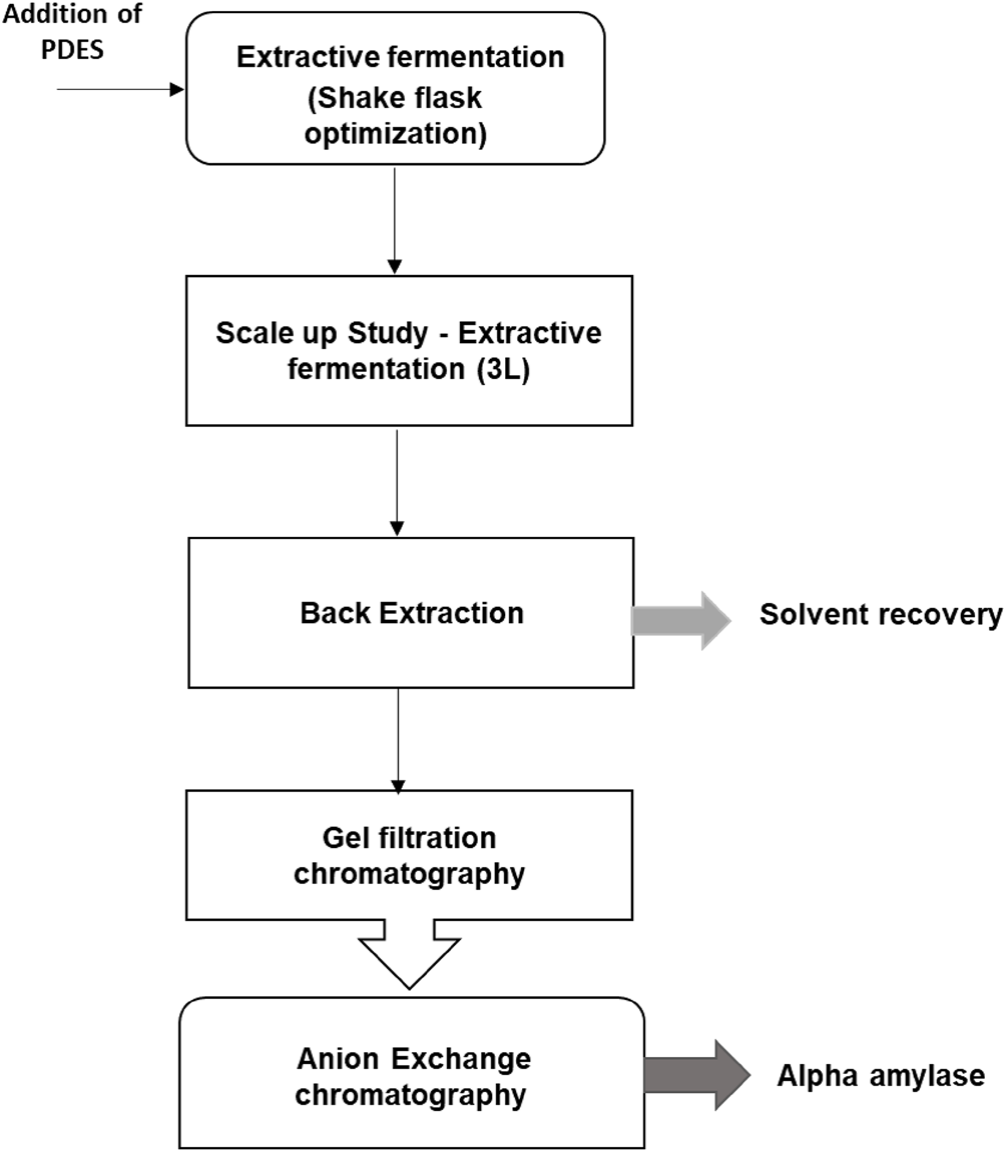
A schematic algorithm of extractive fermentation of thermostable alkaline alpha-amylase using PEG deep eutectic solvents.

The present study involved the extractive fermentation of thermostable α- amylase in a lab-scale bioreactor with PEG-based deep eutectic solvents for a sustainable bioprocess. Five different DES were prepared and utilized for the recovery of amylase. The phase ratio and partition coefficient were evaluated for the best recovery and enzymatic activity. The phase diagram was investigated for all the DES against salt and the tile lie length was determined for effective partitioning of amylase. It was the first attempt to produce and purify thermostable α- amylase in a 3 L reactor at different agitation rates through extractive fermentation using green solvents. The recovery of solvent was achieved through the back extraction method using KCl. The recovered enzymes were purified using gel filtration chromatography followed by DEAE anion exchange chromatography to increase the purity fold. Finally, the effect of pH, temperature, and metal ions sensitivity were investigated. Kinetics of the enzyme activity at different substrate concentrations were also observed. Figure 1 represents the schematic algorithm of the entire study.

## Materials and methods

### Chemicals and media requirements

Glucose (50-99-7), Xylose (58-66-6), Maltose (6363-53-7), Ribose (50-69-1), Lactose (63-42-3), and Polyethylene glycol 400 (9002-88-4) were purchased from Sigma-Aldrich with 99% purity. Starch, Lactose, beef extract, Potassium dihydrogen phosphate, Magnesium phosphate, Ferrous sulfate, and Calcium chloride were procured from Himedia, India with < 95% purity.

*Bacillus simplex was* a laboratory-isolated strain (ON754233) capable of producing amylase enzymes. A loop of isolated culture was streaked on a Leuria Bertini agar plate and incubated at 37 °C for 24 h. When the plate was flooded with iodine, a clear zone surrounded the bacterial growth indicating starch hydrolysis due to the production of extracellular enzymes. The colonies with amylase activity were transferred to the 50 ml Leuria Bertini (LB) broth and maintained at 120 rpm 37 °C^31^. The cultivated apples were procured from local market, Thanjavur, Tamilnadu, India. All of the apples were thoroughly rinsed with distilled water after being disinfected with 1% sodium hypochlorite. The apples were then scrapped and the peels were dried overnight in a hot air oven at 90 °C. The dried biomass was crushed and sieved (0.4 m) before being stored for future use. All the methods were carried out in compliance with the guidelines of International Union for Conservation of Nature (IUCN) and not categorized under IUCN endangered species.

### DES preparation

Five PEG-based deep eutectic solvents (PDES) were obtained by combining sugars and polyethylene glycol (PEG) as hydrogen bond donors and acceptors, respectively (Table 1). Individual HBA and HBD were combined in the molar ratio (1:1) to create the eutectic mixture, which was then heated at 80 °C to create a transparent, homogenous mixture (31). For 45 days, the prepared eutectic mixture stability was monitored in a vacuum at room temperature. With a Rudolf Digital Density Metre (DDM 2910, Rudolf, USA) and a Brookfield Digital Viscometer (LV II +, Brookefield, USA) the density and viscosity of the produced DES were examined.

**Table 1:** Combination of HBAs and HBDs and molar ratio for PDES formation.

DES	HBA	HBD	Molar ratio
PDES 1	Xylose	PEG 400	1:1
PDES 2	Ribose	PEG 400	1:1
PDES 3	Glucose	PEG 400	1:1
PDES 4	Maltose	PEG 400	1:1
PDES 5	Lactose	PEG 400	1:1

### Binodal curve and Tie line determination

The binodal curve is a graphical representation of the two-phase region in a binary mixture. It depicts the temperature and composition combinations at which two phases coexist in equilibrium. By using the turbidometry method, the phase diagram of PDES (60 wt%) against sodium sulfate (40 wt%) was evaluated at a range of temperature between 303 and 313 K, pH 7.2^32^. In a separating funnel, 10 ml of prepared PDES was taken and titrated against aqueous salt solution until the clear solution became turbid (cloud point). The phases were collected individually and the mass of each phase was noted before disturbing the phases with the addition of water. Then to this homogenous mixture, salt solution was again added to form distinct phases. This procedure was repeated until the extract volume was minimal. The concentration of each component in the collected phases was estimated with slight modification studied by Santos et al.^20^

1 $$\frac{{M_{Bot} }}{{M_{Top} }} = \frac{{\left[ {(W \cdot T_{PDES } } \right] - \left[ {W_{PDES} } \right]}}{{\left[ {W_{PDES} } \right] - \left[ {W \cdot B_{PDES} } \right]}}$$

2 $$\frac{{M_{Bot} }}{{M_{Top} }} = \frac{{\left[ {W_{Salt} } \right] - \left[ {W \cdot T_{Salt} } \right]}}{{\left[ {W \cdot B_{Salt} } \right] - \left[ {W_{Salt} } \right]}}$$

where M_Bot_ and M_Top_—mass fraction in bottom and top phase, W.T _PDES_, W.B _PDES_, W.T—weight fraction of PDES in the top phase, bottom phase and total weight fraction, W.T _Salt_, W.B _Salt_, W _Salt_—Weight fraction of salt in top phase, bottom phase and total weight fraction in the system.

In a phase diagram, a tie line (TL) connects the compositions of two coexisting phases. It denotes the state of equilibrium between the two phases at a given temperature and pressure. The distance between the compositions of the two coexisting phases along the tie line is defined as the tie line length. The tie line and slope tie-line (STL) were determined by the following Eq.^33^

3 $$TL = [(W \cdot T_{PDES} - W \cdot B_{PDES } )^{2} + (W \cdot T_{salt} - W \cdot B_{salt } )^{2} ]^{0.5}$$

4 $$STL = \frac{{W \cdot T_{PDES } - W \cdot B_{PDES} }}{{W \cdot T_{Salt} - W \cdot B_{Salt} }}$$

### Extractive fermentation media for the production of α- amylase

EXFEM of α-amylase was carried out in a 250 ml flask for 48 h at 120 rpm. In this experiment, sterile PDES and salt were added to the production media. One day-old culture was transferred to the amylase production media containing starch (12 g/L), Lactose (10 g/L), Beef extract (5 g/L), KH_2_PO_4_ (10 g/L), MgSO_4_.7 H_2_O (5 g/L), FeSO_4_.7H_2_O (0.25 g/L) and CaCl_2_ (0.5 g/L), apple peel (25 g/L), pH 7. The production media went under sterilization at 121 ºC, 15 PSI cooled to room temperature followed by 5% seed inoculation. The inoculated complex media was incubated at 37 °C and 150 rpm for 48 h in a refrigerated incubator shaker (REMI C plus, Remi India)^34^. Then, the system was centrifuged at 8000 rpm for 20 min to get rid of any remaining particles. With a well-defined interface, the DES-rich top and salt-rich bottom phases were separated carefully without disturbing the intermediate layer. The total volume and the individual volume of the two phases were noted. The amylase activity was measured in both phases.

### Optimization of parameters using response surface methodology

The influential parameters such as concentration of PDES, salt, and substrate were determined using central composite design (CCD) based response surface methodology (RSM) for the recovery of α-amylase using the software Design Expert 12. The response mentioned in enzyme activity (U/ml) was determined using a central composite design with three input variables PDES (10–30% w/v), Sodium sulfate (5–20% w/v), and apple peel waste (5–25 g/L). To assess the cumulative influence of individual variables, an absolute design of 20 runs with three center and axial points was created. Three-dimensional response surface curves were generated with two independent factors as variables and response as output. To validate the influence of the factors on the response, a mathematical model was designed with all the designated parameters. All the trials were carried out in triplicates and the results were provided as mean ± SD. And, one way analysis of variance, ANOVA was implemented to assess the significance (*p* < 0.05) and lack of fit.

Every experiment was conducted thrice, and the results were given as mean ± SD. Using SPSS version 21, a one-way analysis of variance (ANOVA) and Tukey's post hoc test were used to determine the significance (*p* < 0.05) level in the data sets. The OFAT experiment data were subjected to principal component analysis using the STATISTICA software (Stat soft. Inc.USA).

**Figure 2:**
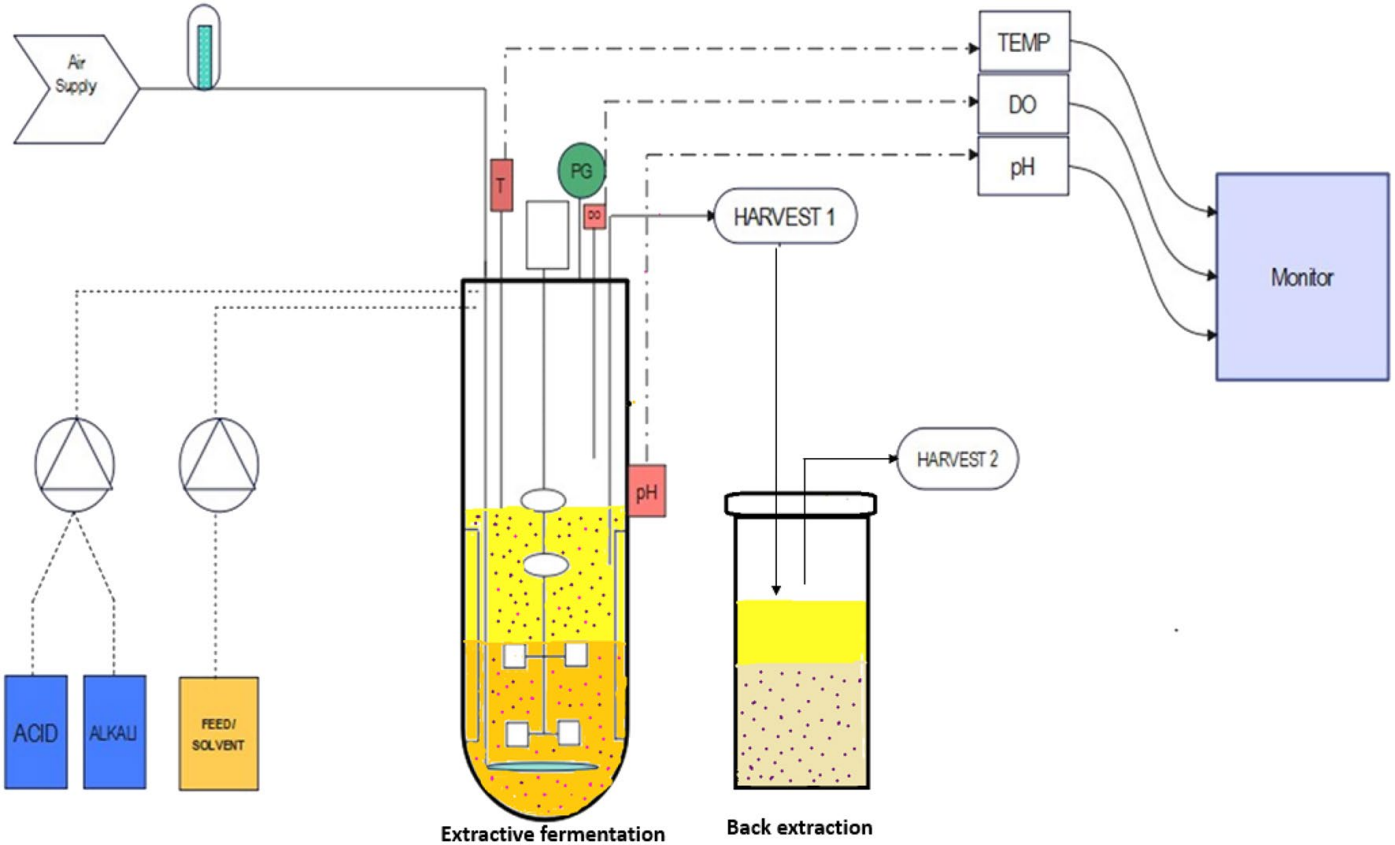
Extractive fermentation of alpha-amylase carried out in 3L benchtop bioreactor with the back extraction unit.

With the optimized parameter in a shake flask level, the fermenter studies were carried out in a 3 L fermenter with a working volume of 1.5 L (Fig. 2). The same shake flask media was utilized in the bioreactor along with DES and salt. The pH of the medium was adjusted to 7. The fermenter along with the extractive fermentation media was sterilized at 121 °C for 15 min. After cooling, one day old culture was transferred to the fermenter and the process was carried out for 96 h. The pH was maintained throughout the process with the addition of 0.1 N HCL/ NaOH. The production rate was studied under different agitation speeds of 120, 150, 200, and 250 rpm with the optimal aeration rate of 1 vvm using a Rushton turbine impeller. With the membrane filter of pore size 0.22 µm, the air was sterilized and sent through a sparger with an airflow meter connected to the system.

### Amylase activity

The individual phases formed were collected and the volume of the DES-rich phase and salt-rich phase were subjected to enzymatic assay. The amylase assay was performed using 3, 5-dinitrosalicylic acid (DNSA) done by Bhatt et al.^35^. 1 ml of 1% (w/v) starch soluble solution with an equivalent amount of enzyme (diluted with 0.05 M phosphate buffer) was incubated for 15 min. 1 ml of DNS reagent was added to the reaction mixture and kept in a water bath for 15 min. Once cooled to room temperature the absorbance was measured at 540 nm to estimate liberated reducing sugars. Glucose was used for standard calibration and the one unit of amylase activity (U/ml) was calculated as the amount of enzyme released per 1 mol of glucose.

### Mass transfer evaluation of extractive fermentation

Both the PDES and salt rich phases were collected carefully without disturbing the phases in a separate vessel and the individual volumes were noted. The phase ratio (P) was calculated by the ratio between the volume of the top phase and the bottom phase.

5 $$P = \frac{{V_{DES} }}{{V_{Salt} }}$$

where P = Phase ratio, V _DES_ = volume of DES-rich extract phase, V _Salt_ = Volume of salt-rich raffinate phase.

Both the collected phases were subjected to enzymatic assay to know the concentration of enzyme distributed between both phases. The partition coefficient (K) was estimated as the ratio of the concentration of amylase activity in the DES-rich extract phase to the concentration in the salt-rich raffinate phase.

6 $$K = \frac{{ C Amy _{DES} }}{{CAmy_{Salt } }}$$

where K = partition coefficient, C Amy _DES_ = Concentration of amylase in the extract phase, and C Amy _Salt_ = Concentration of amylase in the raffinate phase respectively.

The Yield (%) of amylase (Y _Amy_) in the P DES-rich extract phase was estimated using the obtained phase ratio and partition coefficient values as follows

7 $$Y Amy = \frac{PK}{{1 + PK}}$$

Further, the purity factor (PF) was assessed by the ratio of the specific activity of the PDES-rich extract phase to the specific activity in the crude.

8 $$PF = \frac{{S Amy_{DES} }}{{S Amy_{salt} }}$$

where PF = Purity factor, C Amy _DES_ = Concentration of enzyme in DES rich extract phase C Amy _Crude_ = Concentration of enzyme in crude.

### Back extraction

The back extraction method performed the recovery of amylase from the solvent-rich phase. The solvent-rich phase was transferred to a sterile flask with an equal volume of fresh aqueous salt solution of potassium chloride (0.1 to 0.5 M) added. The Vander wall’s force destabilized between the DES and enzyme, leading to the phase separation. The amount of active enzyme and corresponding DES was determined as detailed by Ramya et al.^32^. During back extraction, the enzyme transfers from the PDES-rich phase to the aqueous phase. This back extraction process is accounted for by the new partition coefficient (K') given by the following Eq. (9). The recovered DES (R) was calculated by the following equation.

9 $$K^{\prime } = \frac{1}{K }$$

10 $$R = \frac{PDES\,\, recoverd}{{Initial\,\, mass\,\, of\,\,PDES }} \times 100$$

where K′ = Partition coefficient for back extraction process, K = Partition coefficient of extractive fermentation. PDES recovered = the mass of the PDES recovered at the end of the back extraction. The initial mass of PDES = a mass of PDES present in the system before back extraction. This formula calculated the PDES recovery as a percentage of the PDES recovered from the initial mixture. It provides a quantitative measure of the efficiency of the back extraction process. The higher the percentage of recovery the more efficient the process of extracting and recovering the DES.

### Purification of α-amylase

The extract from the salt-rich phase was loaded on 5 ml Sephadex G-15, Size Exclusion chromatography (Akta Pure) for desalting and the separation of the target molecule based on the molecular size. 20 mM Tris buffer at pH 7 was utilized for the column equilibration. First, the column was equilibrated with the equilibration buffer where the equilibration volume is 5 to 10 times the column bed volume (CV). The flow rate was set to 2.5 ml/min and made sure that the buffer cover the entire resin bed. Once the column was equilibrated, the sample was loaded at a 1 ml/min flow rate. The sample was diluted with the equilibration buffer with a minimum loaded volume of 0.5% to 2% of the total column volume (CV). If the resolution between the target protein and the contaminants is to be eliminated, even higher sample sizes may be suitable. The sample can be concentrated before for better resolution and to avoid band broadening.

#### Ultra purification by DEAE anion exchange chromatography

The eluent fraction from the Sephadex G 15 was loaded into the DEAE anion exchange 5 ml column using GE AKTA Pure. Based on interactions between charged molecules and ion exchange resin, ion exchange chromatography is used to separate and purify charged molecules. The column was equilibrated with 20 mM Tris–HCL, pH 7 buffer. The sample was loaded through the sample port which was passed to the column. Next, the washing step was carried out with the same equilibration buffer to eliminate any unbound or weakly bound impurities. The salt concentration in the wash buffer is typically lower than in the elution buffer. Elution was accomplished by the elution buffer 20 mM Tris–HCL, 0.5 M NaCl at pH 7. Due to ionic strength disruption, the target molecule was eluted from the column using a gradient of increasing ionic strength. This salt competes with proteins to bind to the DEAE resin, causing them to be eluted from the column. Finally, the absorbance of the effluent at an appropriate wavelength was monitored and the chromatogram was absorbed and recorded. Then these fractions were collected and then examined with SDS PAGE for its purity and identification of our target molecule. As a final part, the column was thoroughly cleaned using the appropriate solvents to regenerate it. By doing so, impurities have been removed and the ion exchange groups are renewed for future usage.

### SDS PAGE electrophoresis

All fractions obtained via extractive fermentation and purification underwent SDS PAGE electrophoresis. PAGE was performed using a BioRAD mini protean system. 5% stacking gel and 12% separating gel were prepared and the samples along with a high molecular weight marker were loaded into the well. The gel was run at 100 V till the sample had run through three-fourths of the gel. Further, the gel was removed carefully from the plates and stained. Overnight coomassie strain was done followed by de-staining.

### Effect of temperature and pH on α-amylase activity

The amylase activity of the purified enzyme of the PDES-rich phase was subjected to temperature stability. The temperature effect was determined by incubating the enzyme at different temperatures varied from 20 to 80 °C for 60 min. The aliquots were cooled to room temperature to ensure effective refolding of the enzyme and the residual amylase activity was measured in the DES-rich phase. Likewise, the pH stability of the enzyme was calibrated using distinct buffers. 0.1 ml of enzyme was mixed with 2 ml of 0.1 M of citrate—phosphate buffer (pH 5–7), Tris—HCl (pH 7–9) and allowed to incubate for 60 min. Finally, the amylase activity of each aliquot was evaluated.

#### Effect of metal ions and inhibitors on α-amylase activity

The amylase activity of the purified enzyme was evaluated in the presence of different metal ions and inhibitors. The partially recovered enzymes were in 1 M Tris- HCl buffer was exposed to various metal ions such as ions potassium (K^+^), calcium (Ca^2+^), Magnesium (Mg^2+^), copper (Cu^2+^), and zinc (Zn^2+^), cobalt (Co^2+^) and metal inhibitors such as EDTA and SDS at 5 mM concentration. All mixtures were incubated at 37 C for 60 min to measure the amylase activity.

#### Kinetic parameters

Using a double reciprocal Lineweaver–Burk plot (Eq. 11), different concentrations of starch (0–10 mM) in phosphate buffer were used to estimate the Michaelis–Menten constant (Km) and reaction rate (Vmax). The non-linear Michaelis–Menten equation was used to fit the data with the observed amylase activity for different concentrations of the substrate.

11 $$\frac{1}{V} = \frac{{K_{m} + \left[ S \right]}}{{V_{m} \left[ S \right]}}$$

## Results and discussion

### PEG-DES characteristics

**Figure 3:**
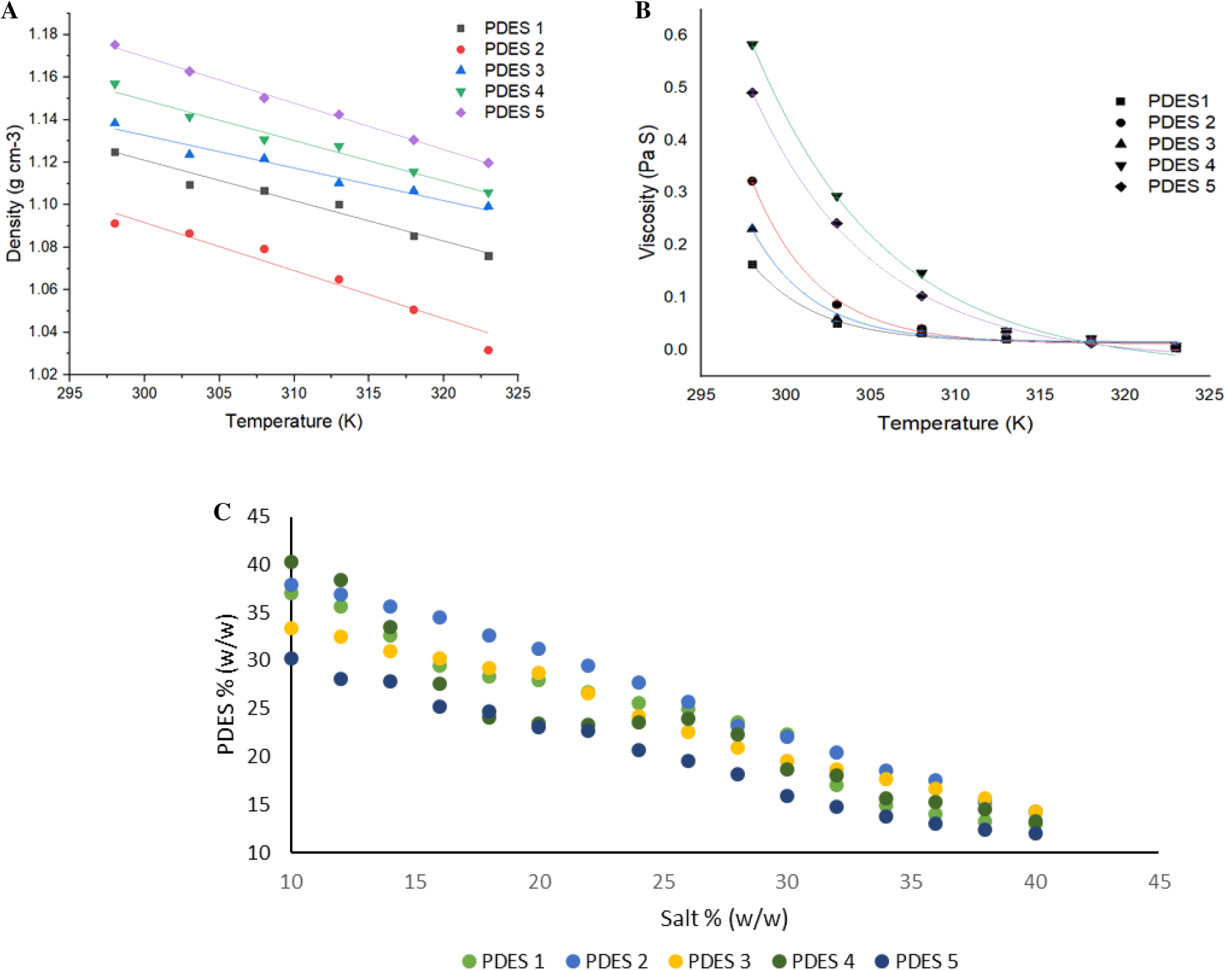
(**A**) Density (**B**) Viscosity. Effect of temperate on PDES at an interval of 298 to 323 K. (**C**) Binodal phase diagram between the PDES and salt of different concentrations. (Mean values ± standard error of three replications).

The density and viscosity of all PEG-based DES (PDES) were measured as a function of temperature in the range of 293.15–323.15 K Fig. 3A. The density of all PDES was found to decrease in a monotonous way as the temperature increased. This could be due to a significant increase in the entropy of the homogeneous system, which influences the hydrogen bonding pattern in the PDES, reducing its density as temperature increases. This linear behavior has consequences for the extraction process, especially if temperature changes occur during the extraction process. Higher-density DES has a low extraction capacity, whereas lower-density counterparts have a high salting capacity reducing the phase volume of the extract. As a result, PDES with a moderate density with less influence over the aforementioned temperature range was chosen for effective amylase recovery. The highest density was observed for DES 5 at 1.197 g c^−3^ and the lowest at 1.076 g cm^−3^ at 323 K. The density order at both extremes of temperature was found to be PDES 2 < PDES 1 < PDES 3 < PDES 4 < PDES 5. The linear density-temperature relationship may have an impact on this solubility behavior and the solubility may change linearly with temperature, resulting in temperature-dependent extraction efficiency^36^. Also, temperature control is critical for maintaining consistent extraction conditions and reproducibility which could be useful in designing temperature control strategies during the extraction process^37^. Glycol-based DES with moderate density was observed to be effective in the extraction of flavonoids studied by Zhao et al. and proved DES as a promising green solvent in the extraction process^38^.

DES viscosity can be extremely sensitive to temperature and the specific components chosen in the preparation of DES. Furthermore, the viscosity of the mixture may vary depending on the concentration of the components. During the extraction process, viscosity influences the reaction kinetics and mass transfer rate and the reaction rate slows down with higher viscosity. The higher viscosity was observed for PDES 4 (0. 67 Pa.S) and lowest for PDES 1 (0.012 Pa.S). The order of viscosity of PDES as follows PDES 1 < PDES 3 < PDES 2 < PDES 5 < PDES 4 (Fig. 3 B). Viscosity is frequently temperature-dependent and decreases with an increase in temperature. The strength and number of hydrogen bonds formed between the HBA and HBD in the DES can have a significant impact on its viscosity. Higher viscosities are associated with stronger hydrogen bonding^39^. The less viscous DES is preferred for ease of mixing and mass transfer in extraction processes, whereas higher viscosity DES may be used in applications requiring greater solute retention or immobilization. A similar investigation has been done for the long-term extraction of phenolic compounds with novel (DES) with less viscosity^40^. Hence both the density and viscosity are the critical components of DES rheological characterization. This information about DES reveals the flow behavior, shear stress, and shear rate dependence^41^. This knowledge is essential for designing process equipment, selecting appropriate pumps or mixers, and comprehending the flow properties of DES during extraction or storage.

### Influence of tie lie length on α-amylase partitioning

The binary phase diagram of the corresponding PDES (Fig. 3C) shows the impact of HBA and HBD on two-phase formation. The area below the binodal curve represents the single-phase region, and the region above it represents the biphasic region. Thus, the binodal curve is a boundary line between them. The cloud point method was used to obtain binodal data for PDES against sodium sulfate solution^42^. It was observed from the study that the PDES with high density have a deep binodal curve that consumes more salt, whereas less density PDES consume very little salt with shallow curve. As a result, PDES with moderate density has sufficient salting-out volume, resulting in a large biphasic region. Due to the entropy changes in the system, PDES and salt combination influence the stability of the biphasic system. It was observed that the exclusion volume was increased with the increase in the mass of PDES utilized. Also, there was a large room for glucose and PEG due to higher interaction with the salt and the water content in the media compared to other DES. The order of binodal area for all the five PDES corresponding to the sodium sulphate solution followed a pattern PDES 2 < PDES 1 < PDES 3 < PDES 4 < PDES 5. A similar pattern was observed for the amylase partitioning into PEG 6000 and Na_2_SO_4_ with the highest void space with higher polymer-salt-water interaction^20^. Also pH of the system influenced the shift in the binodal curve and it was observed there was a shift in the binodal curve towards the lower PEG and salt concentration in the partitioning of amylase with increasing pH was investigated by Nascimento et al.^43^. The mass of salt added also influence the phase partitioning and lead to high partition values. The denser PDES required more salt with a deep binodal curve whereas less dense PDES required a small amount of salt with a narrow curve. With sodium salt, the recovery may be increased due to the affinity between Na^+^ with DES and the partition would be enhanced compared to phosphate and other salts^44^. The selection of salt is critical in cloud point extraction as it favors the formation of distinct phases and the salting-out effect has a significant impact on the volume ratio of the solvent and salt phases^45^. Also, the sulfate salt possesses moderate ionic strength and influences the phases by increasing the phase volume and cloud point followed by the Hoffmiester series implying that higher valence anion have better salting-out^46^. Table 2 shows the relationship between TLL and STL of GDES along with the sodium salt. The TLL and STL values for PDES 3 were 42.98 and 1.32, indicating a possibility of higher active amylase recovery. This resulted in a transfer of DES into the top phase and salt shifted to the bottom phase. When the glycol-based DES was utilized for the recovery of fibrinolytic protease, TLL showed 62.43 with a slope of 1.622 and was found to be thermodynamically favorable partitioning of protease into DES-rich phase^32^. Chen et al. investigated the PEG-based DES dissociation in water along with the surface tension and surface thermodynamics and observed that DES has a dissociation tuning point with water which was not found with any other solvents^47^. The larger inclination of tie lie length occurred due to the higher solubility of the DES to form distinct phases. Moreover, the tie line length in the phase equilibrium calculations, particularly in binary phase diagrams represents the compositions of two coexisting phases. Although the length of the tie line does not directly influence partitioning, it could be a useful parameter in understanding the partitioning of components in a mixture. The phase separation becomes sensitive as the tie-line length decreases, and it is complex for a phase composition close to the plait point^48^.

**Table 2:** Tie-lie length, Slope tie line for the GDES and salt system with the mass fraction of DES and salt in the top and bottom phase.

Solvent/Sulphate Salt	TLL	STL	Top phase (% w/w)		Bottom phase (% w/w)	
			M DES	M Salt	M DES	M Salt
PDES 1	34.47	2.34	31.56	4.485	0.88	18.02
PDES 2	32.85	1.86	29.29	4.82	0.845	20.01
PDES 3	42.98	1.32	39.79	1.59	0.095	17.7
PDES 4	37.46	1.1	35.02	3.69	0.295	17.72
PDES 5	34.2	2.56	30.94	4.48	0.595	20.79

### Optimization of α-amylase through response surface methodology

**Figure 4:**
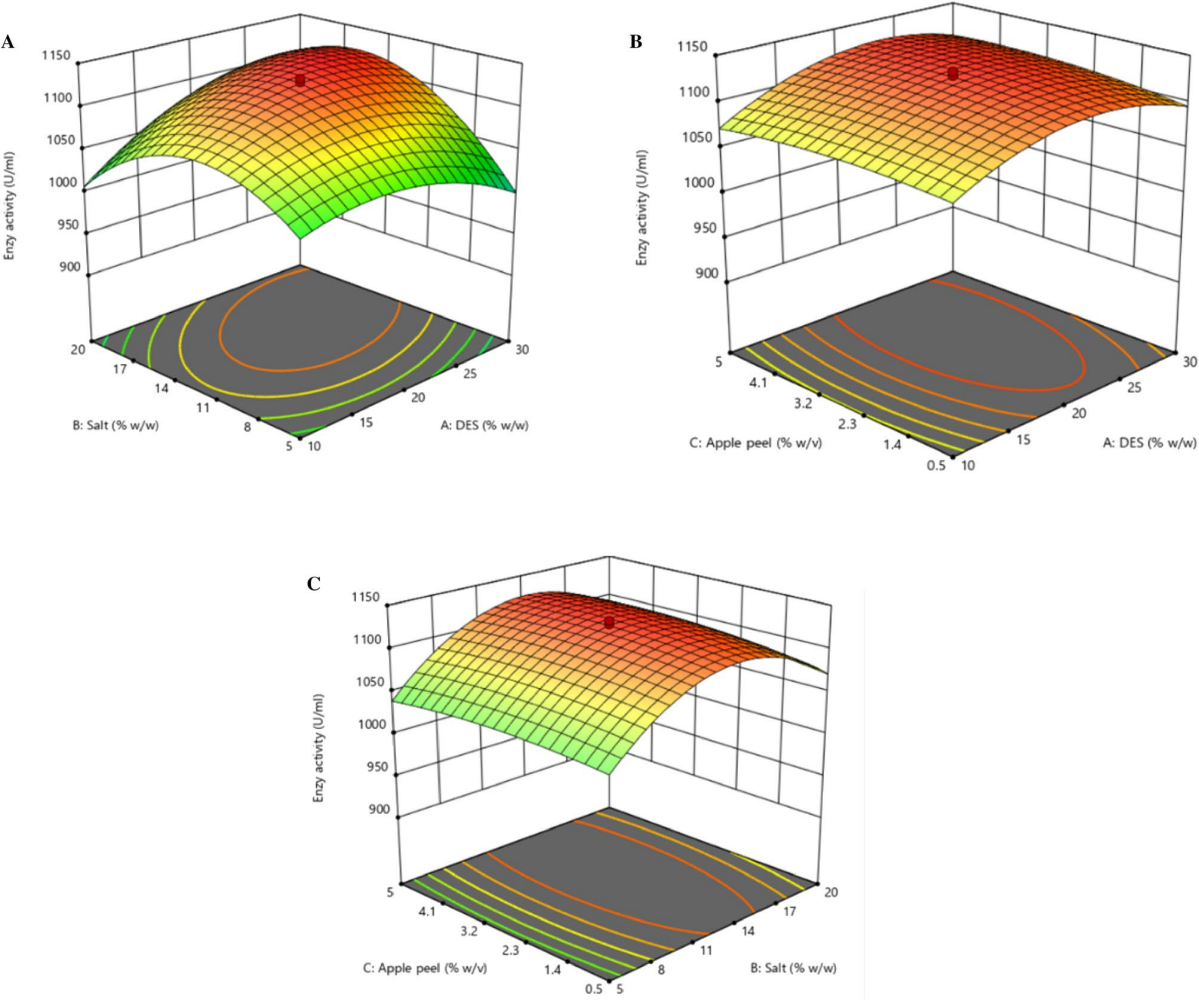
RSM for α amylase production from *Bacillus simplex* with influential parameter with response to enzyme activity (U/ml). (**A**) The interaction between PDES and salt concentration. (**B**) Apple peel and PDES concentration (**C**) Apple peel and salt concentration.

The influential parameters for the production of thermostable α-amylase from the lab-isolated strain *Bacillus simplex* (ON754233) were by CCD-based RSM using Design software expert 12. The parameters selected were PDES concentration (A), salt concentration (B), and apple peel (C) with the other compositions kept constant (Fig. 4). 20 trials were run using various combinations of A, B, and C with the amylase activity as a response. The design results were used to develop a second-order polynomial quadratic regression equation for amylase activity which was shown below and the model was found to be significant with the P value < 0.0001.

13 $$R1 = 1124.37 + 15.26 A + 18.91 B + 3.30 C + 33.12 AB + 4.76 AC + 4.28 BC - 30.98 A^{2 } - 59.16 B^{2} - 7.49 C^{2}$$

The result witnessed the PDES and salt concentration greatly influenced the recovery of amylase into the PDES-rich phase while the apple wastes had a moderate effect compared to the phase-solving components in the broth. As the apple waste has less effect on the enzyme activity, the influential parameters such as PDES and salt played a major role in extracting the protein into PDES rich phase. A similar result was observed in the production of fibrinolytic protease, where increasing the brewery liquid waste (carbon source) had no significant effect on the recovery of protease^32^. Also the increase in the carbon source has little influence on enzyme recovery. The current model was predicted for R^2^ value, adjusted R^2^, predicted R^2^, and lack of fit. The lack of fit “F-value” was found to be 4.12 showing “Non-significant” with *p* value 0.217. This result “Non- significant” lack of fit with the model for the estimation of recovery of the enzyme. From Table 3, it was evident that A, B, and AB values were significant with *p* < 0.001 and F values found to be 8776.8 (AB). This result demonstrated that the significant parameters were DES and salt compared to the apple waste. Further, the p-value ascertained the correlation between the significance of the model parameters. When the DES and salt concentration has been increased to 20%w/v and 12.5% w/v, the enzyme activity was increased to 1126.78 ± U/mL and started decreasing when increased or lowered beyond that concentration. This is evident that the enzyme infiltrates either into salt phase or into the intermediate layer. So the concentration of the phase forming solvents were very important in the extraction of enzymes into the DES rich phase. This result was similar to the investigation of amylase production during optimization of various parameters and the lack of fit value was found to be 3.32 with *p* < 0.0001 and R^2^—0.9998^49^. The obtained values for the current CCD models were R^2^—0.9971, adjusted R^2^—0.9968, and predicted R^2^—0.9960.

**Table 3:** Amylase activity ANNOVA table resulting from by response surface quadratic model.

Source	Sum of squares	df	Mean square	F-value	*P* value	
Model	73,937	9	8545.56	133.45	< 0.0001	Significant
A-DES	3181.13	1	3181.13	49.66	< 0.0001	
B-Salt	4883.41	1	4883.41	76.24	< 0.0001	
C-Apple waste	149.08	1	149.08	2.33	0.1581	
Ab	8776.8	1	8776.8	137.02	< 0.0001	
AC	181.07	1	181.07	2.83	0.1236	
BC	146.55	1	146.55	2.29	0.1613	
A2	13829.51	1	13,829.51	215.89	< 0.0001	
B2	50440.57	1	50,440.57	787.43		
C2	809.15	1	809.15	12.63	0.0052	
Residual	640.57	10	64.06			
Lack of fit	566.49	5	113.3	4.12		Not significant
Pure error	137.5	5	27.5			
Cor Total	77577.57	19				

R^2^ = 0.9971, Adjusted R^2^ = 0.9968, Predicted R^2^ = 0.9960.

### Extractive fermentation of α-amylase in a bioreactor

**Figure 5:**
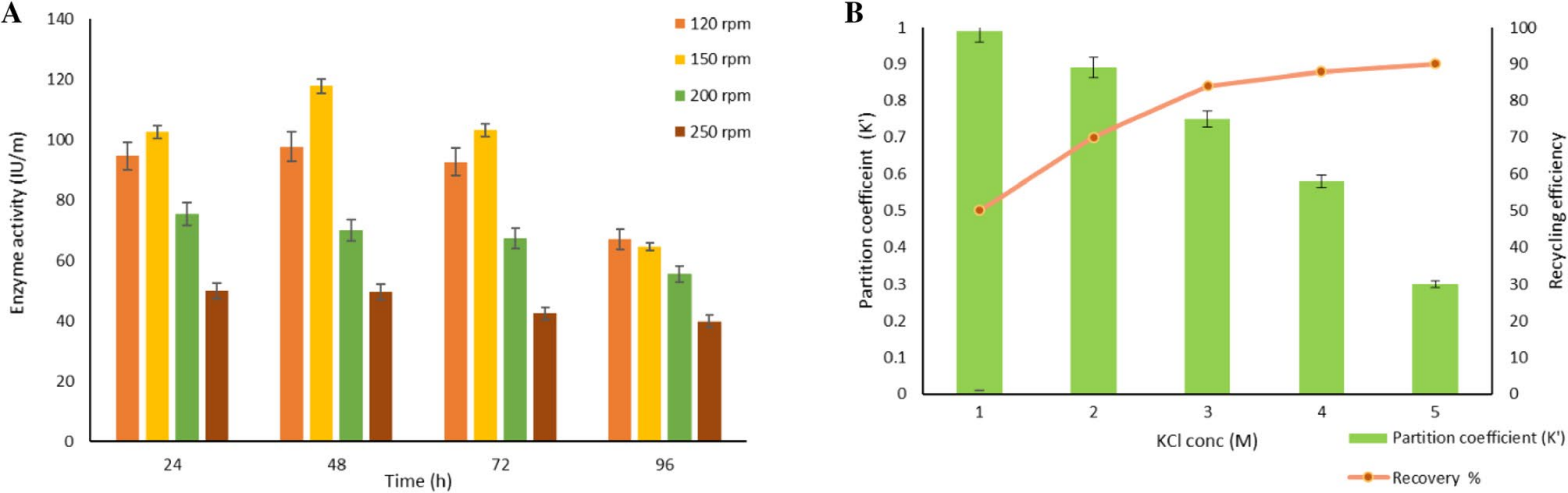
(**A**) Effect of agitation rate on enzyme activity at different time intervals for the production of α-amylase at optimum temperature 37 °C, pH 7.2, and aeration rate 1 vvm after 96 h. (**B**) Back extraction of amylase into salt-rich phase. The partition coefficient and the recovery of DES (%) concerning the varying concentration of KCl. At 0.5 M KCl, the recovery of DES was around 92%.

The extractive fermentation of α-amylase was carried out in a 3 L bioreactor with the optimized parameters in a batch mode. The phase-forming solvents and the media were sterilized and the seed culture was inoculated followed by a 96 h batch process. Once the amylase production started the enzyme was partitioned into a PDES-rich phase based on its hydrophobicity. The affinity towards the PEG 400: Glucose with the enzyme was more compared to other DES and the extraction efficiency was found to be 92.4% with the highest partition coefficient value of 3.59. The enzyme activity was found to be 2200.13 ± 1.02 IU/ml during extractive fermentation with apple peel as a source. This result was compared to the amylase activity of conventional fermentation during 48 h of incubation with banana peel as substrate reported as 821.33 ± 0.57 IU/mL^50^. As DES is very task-specific, it can be tailor-made according to our requirements, this PEG-based DES was prepared for the effective recovery of amylase during fermentation^51^. The smaller molecular weight of PEG influences the extraction of enzymes towards the DES phase rather than partitioning into the salt phase. A similar investigation was observed in the extraction of RNA with less molecular weight PEG combined with longer alkyl chain quaternary ammonium salts^52^. Further, the reactor studies were carried out at different agitation rates such as 120, 150, 200, and 250 rpm at a constant temperature with the aeration rate of 1 vvm, and its corresponding enzyme activity was observed (Fig. 5A). The enzyme activity was found to be optimal at 150 rpm, 48 h, and the cell growth was optimum till 150 rpm, and beyond that speed, the amylase activity was lowered due to the high shear rate. At 200 rpm the enzyme activity was reduced to 700.65 ± 0.09 IU/mL. When the agitation speed was increased to 250 rpm the shear rate became detrimental to the cells the production was restricted and the enzyme activity was lowered^53^. During fermentation, agitation initiates mixing and shear that allows the effective transfer of oxygen, nutrients, and heat in the broth through the dispersion of air bubbles favoring the organism to utilize the nutrients completely. The Rushton turbine impellor with moderate agitation has a strong ability to break up bubbles and provide the oxygen supply for the cells to grow resulting in enhanced enzyme activity. Further increase in agitation speed, excessive bubble crested foam suppressed the nutrient supply and mass transfer rate in the media. On the other hand, the low agitation speed increases the viscosity of the broth resulting in a low mass transfer rate, and the optimal speed was observed as 200 rpm to produce glycoprotein GP-1 studied by Zhou et al.^54^.

### Back extraction and recovery of PDES

One of the major advantages of DES extraction is that it can be easily recovered and recycled compared to other conventional solvents. Once the top phase was transferred into a separate vessel, it was mixed with KCl by varying its concentration. The system was agitated mildly to ensure that the salt was evenly dispersed to promote interaction between the enzyme and salt. The system was kept constant until two distinct phases formed where the interaction between PDES and salt weakened and enzymes partitioned towards the salt phase. The optimum KCl concentration was found to be 0.5 M and 90% of PDES was recycled. When the partition coefficient is less than one, it indicates that the protein is being transferred to the salt phase (Fig. 5B). When the salt concentration was increased the salt reduced the solubility of protein and started transferring to the aqueous phase resulting in a lower top phase. The obtained results were similar to the back extraction of RNA when Na_2_SO_4_ was added and the recovery of Peg based DES was found to be 92%^52^. This is one of the major advantage of using DES in the extraction process, where most of the protein get filtered into the DES rich phase on selecting the suitable DES which should not degrade the protein. This results in the reduction of one purification step as the solvent was recovered for almost 80 to 90%, the sample could be directly loaded into the chromatographic column without any prior purification step.

### Purification of α- amylase

**Figure 6:**
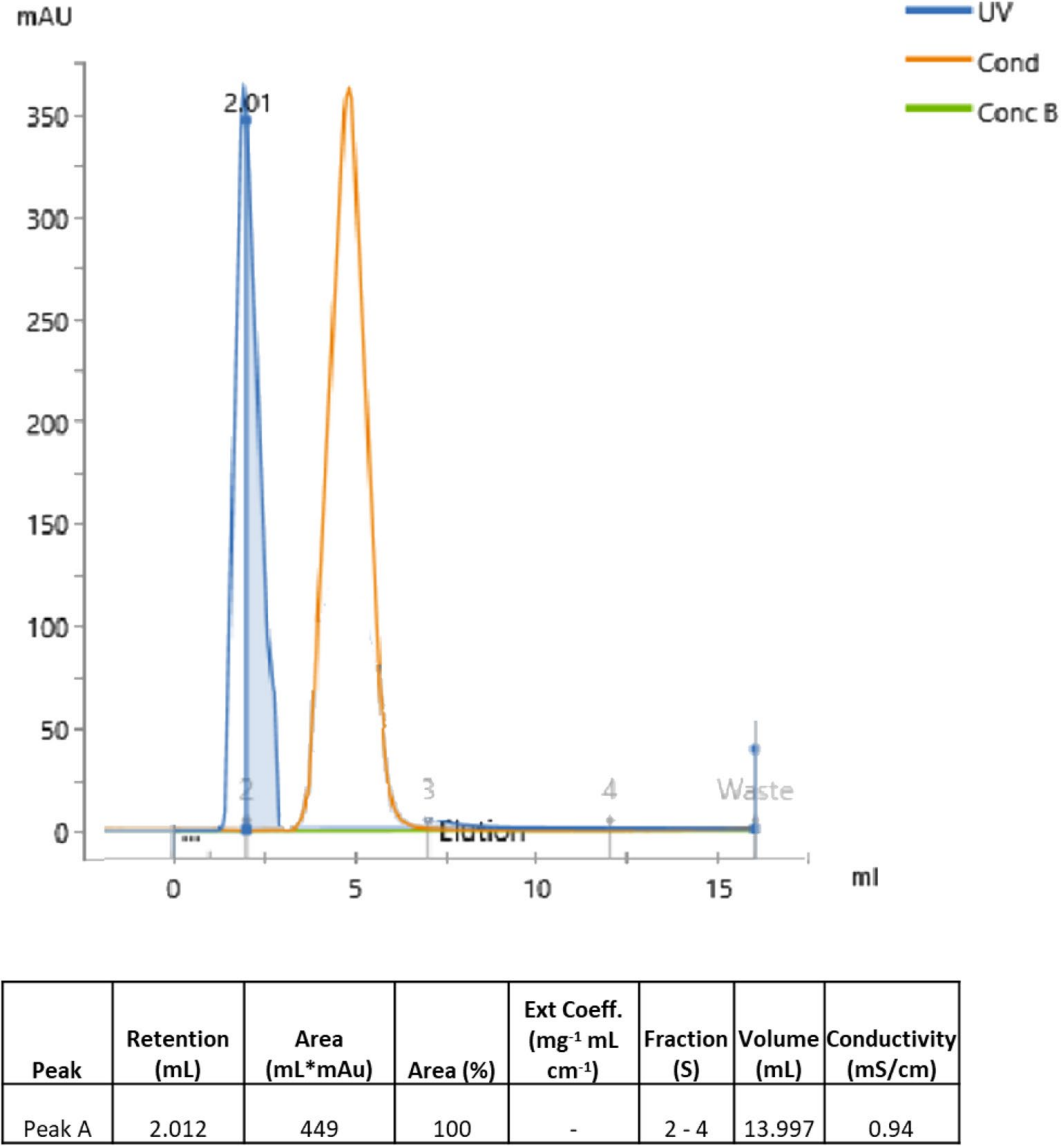
Gel filtration chromatography—separation of thermostable α—amylase with the elution peak of around 350 mAu. The salt from the back extraction sample was removed which was visible as the conductivity peak.

The partially purified sample was loaded into the Sephadex G-15 column for the buffer exchange, desalting, and separation of the target protein. The column was equilibrated followed by sample loading at 1 mL/min. once the sample was loaded into the column the sample passed through the porous matrix of Sephadex. Again the equilibration buffer was passed to transport the proteins through the column by maintaining the integrity of the molecules. The larger molecular weight eluted faster compared to the smaller molecules. A single sharp peak was observed in the chromatogram (Fig. 6) with a total volume of 13 mL and, a retention volume of − 2 ml with the UV range of around 350 mAu. The area under the curve was calculated from the retention volume and peak height was observed to be 449.0. The other protein with the least molecular weight was eluted and was also observed in the chromatogram with the least enzyme activity (data not present here). During the process, the salt was removed from the sample and the final conductivity was lowered to 0.94 mS /cm the enzyme activity was found to be 1680.31 ± 0.5 IU/mL with around 81%.

The collected peak from gel filtration chromatography was loaded into DEAE sepharose anion exchange chromatography. Once the column was equilibrated the sample was loaded into the column followed by gradient elution. The eluted peak was collected and subjected to enzymatic assay. The final recovery was around 90 % with a purity fold of 33 (Table 4). The amylase activity was found to be 1477.58 ± 1.16 IU/mL. A similar study was investigated for the purification of alpha-amylase from *Aspergillus flavus* using anion exchange chromatography with a purity fold of 2.55 and recovery of 11.3 % during conventional fermentation and purification methods^55^. Another study showed 51 purity fold with DEAE cellulose column chromatography for α- amylase from *Bacillus licheniformis* with 30 % recovery^56^.

**Table 4:** Recovery table of thermostable α-Amylase with the recovery (%) and purity fold.

S. No	Steps	Volume (ml)	Total protein (mg)	Enzy activity (IU/mL)	Specific activity (U/mg)	Recovery %	Purity fold
1	Extractive fermentation	1200	4209.6	2200.13	627.175	100.00	1.00
2	Back extraction	950	645.05	1940.221	2478.51	88.19	3.95
3	Gel filtration n	10	2.565	1680.312	6986.63	76.37	11.14
4	Ion exchange	5	0.495	1477.583	20779.6	67.16	33.13

### Molecular weight determination by SDS PAGE electrophoresis

**Figure 7:**
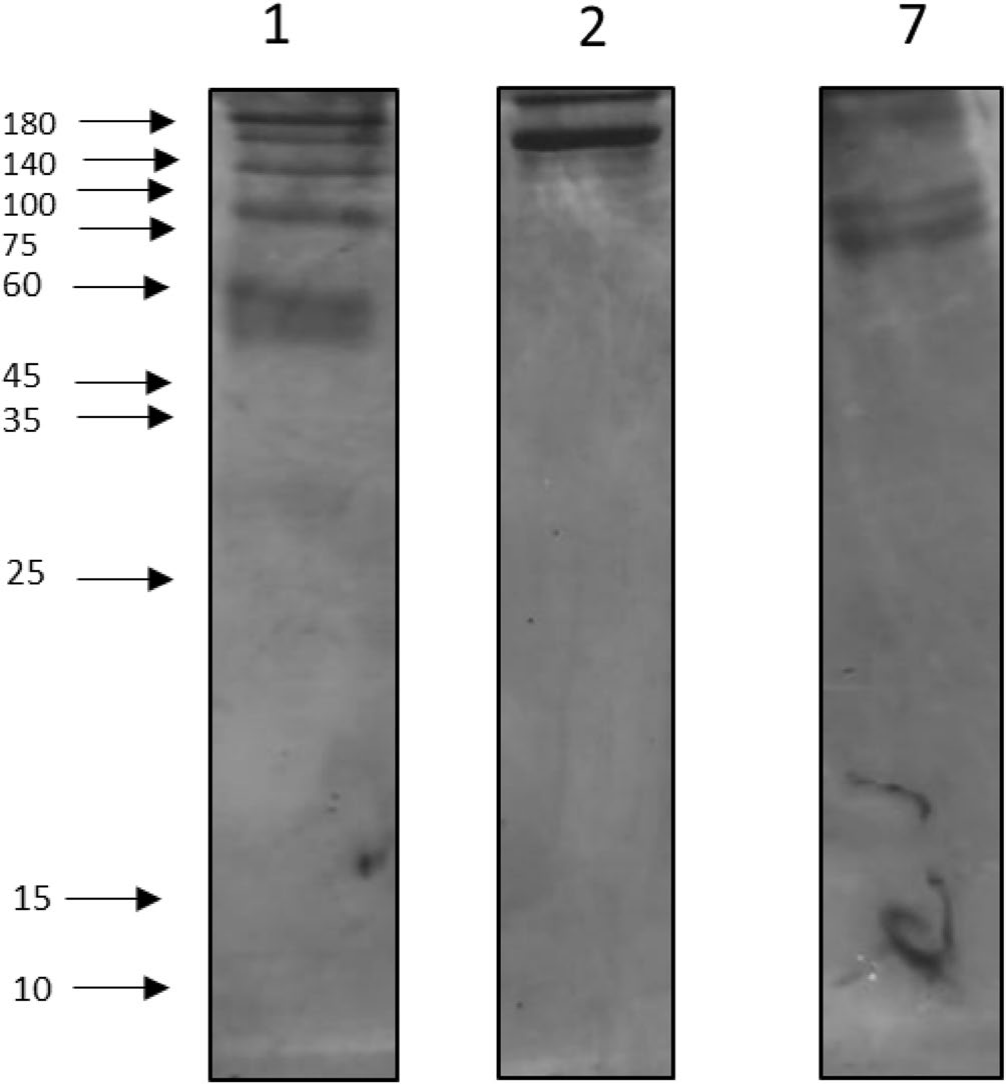
SDS PAGE electrophoresis. Lane 1—High molecular weight marker (180 to 10 kDa), Lane 2—GFC elute with a clear single band, Lane 7—Extractive fermentation crude with other impurities. The gel with all the lanes was given in the supplementary file.

The aliquots from the partially purified enzyme from extractive fermentation and elute from gel filtration chromatography were loaded into the SDS PAGE electrophoresis unit and the molecular weight and the purity was determined. The bands were examined under a UV transilluminator (Fig. 7). The high molecular weight marker was loaded into lane 1, elute fraction collected from GFC in lane 2, followed by partially purified enzyme in lane 7. The image of full gel was given in the supplementary file. The band was identical to a protein ladder and identified as 120 kDa approximately. The clear single band clearly showed that the enzyme purity was enhanced through extractive fermentation and the molecular weight was observed to be in the range of 100 to 140 kDa. Mehta et al. also reported that the majority of bacterial amylases had molecular weights ranging from 10 to 210 kDa, with only a few having low molecular weights^57^.

### Effect of temperature, pH, and metal ions on α- amylase

**Figure 8:**
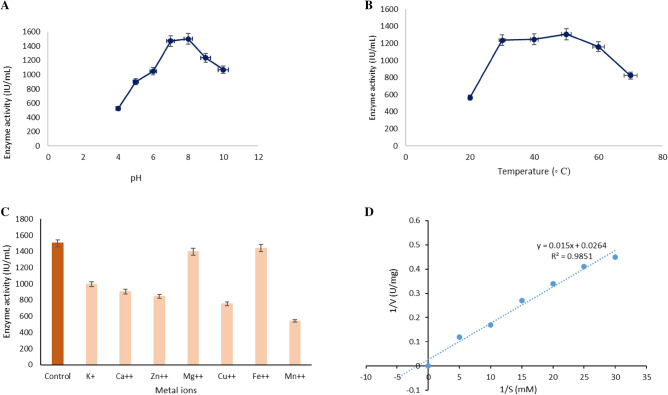
Effect of pH, temperature, and metal ions on amylase activity. (**A**) Effect of enzyme activity at different pH from 4 to 10. The stability of the enzyme was more at an alkaline pH of around 7–8. (**B**) Effect of temperature on enzyme activity in the 20–80 °C range. (**C**) Effect of enzyme sensitivity on different metal ions and compared with control incubated in the absence of metal ions. (**D**) The amylase activity plot against different substrate concentrations using the linear regression method Lineweaver–Burk plot to find out the amylase kinetics K_m_ and V_max_ values.

The optimum pH, temperature, and metal ions of the amylase produced through extractive fermentation from *Bacillus simplex* were evaluated using different buffers (pH 5–9), temperatures (30–100 °C), and metal ions under incubation time of 60 min. When the enzyme in Tris HCl buffer of 7–9, the optimum pH was found to be at 8 with high enzyme activity revealing that the enzyme was active and stable in alkaline conditions. This alkaline amylase remains active till pH 9 and starts losing its activity beyond pH 10. When subjected to acidic pH the enzyme stability was greatly reduced and less activity was observed (Fig. 8A). This result was similar to the investigation on extractive fermentation of protease from *Aspergillus tamarii* using a PEG-salt system and found the enzyme was stable over a broad pH range of 7–10^58^. When the temperature profile was studied between 20 and 80 °C, the amylase activity was observed to be optimum at 60 °C (Fig. 8B). After exposure for 60 min, the amylase activity remains active between 40 and 60 °C showing that the purified amylase was thermostable. Beyond 60 °C, the purified amylase starts losing its activity. This observation was compared to the extractive fermentation of protease with an optimum temperature of 60 °C when exposed for 180 min^59^. Also, the amylase activity was stimulated and observed activity of 90% when exposed to the Mg + + followed by Fe +  + , and the activity was observed to be 87% confirming the stability against these salts. The activity was reduced to 76% when exposed to Cu + + showing that the enzyme was little sensitive to copper ions and the enzyme was sensitive to Zn +  + and Cu +  + as the maximum inhibition observed with these ions (Fig. 8C). These findings were in contrast to the protease, when exposed to Mg + + ions, the enzyme activity was stimulated followed by Ca +  + , Fe +  + investigated by Sales et al.^25^.

### α-Amylase kinetics

The specific enzyme activity was calculated against different substrate concentrations to estimate Km and Vmax using linear regression of the Lineweaver—Burk plot. The different concentration of starch was plotted against the amylase activity and the Km and Vmax was calculated. From Fig. 8D, the kinetic constants were evaluated. The Km and Vmax were found to be 0.00039 mM and Vmax 37.87 U/mL showed affinity between the enzyme and substrate. A low Km value with a high Vmax value showed that the amylase produced through extractive fermentation could achieve its maximum velocity at lower substrate concentration. Similarly, a smaller Km value of 0.55 mM was obtained during the solid-state fermentation of amylase with a similar investigation done during the solid-state fermentation of amylase and achieved maximum catalytic effect^60^.

## Conclusion

The present study investigated the extractive fermentation of thermostable alkaline amylase using PEG-DES from *Bacillus simplex* ON754233 with apple pomace as a carbon source in a batch mode. For the first time, we made an attempt to produce and purify the thermostable alpha amylase through extractive fermentation using DES. The amylase was partitioned into the PDES- rich phase evident that the protein extraction into the DES phase was favored with less excluded volume compared to the salt phase. Through back extraction with KCl, the solvent was recovered around 88% from the PDES-rich phase which could be recycled or reused. To confirm the feasibility of extractive fermentation in large scale, the process was scaled up to 3L custom made bioreactor with the amylase activity of 1940.22 IU/mL. Further, the recovered enzyme was purified using gel filtration column followed by anion exchange column and the purity fold was enhanced to 33 times. The Vm and Km values were calculated using the Lineweaver–Burk plot as 37.87 U/ml and 0.000396 mM. These values provide critical insights into the kinetics and behavior of enzymes, which is important in biochemical studies, drug development, and understanding cellular functions. As a result, PEG DES investigation in extractive fermentation holds a promising technology on the industrial scale with minimal unit operations. Additionally synthesis of DES is very simple and could be recovered, recycled, and reutilized having a great impact on the overall cost of production. Utilizing process integration technology of simultaneous recovery during fermentation with green solvents will improve the sustainability of any bioprocessing sector by enhancing the yield with less accumulation of byproducts. One of the major advantages of this technology is that we can design the reactor and solvents according to the target molecule without compromising any standard operating procedures. Although extractive fermentation is well known in the biofuel production, the enzyme production on a large scale is inadequate. Despite the fact that EXFEM has been extensively studied, scaling up remains uncertain owing to ignorance, reactor configuration complexity, and solvent selectivity. The need for sustainable and affordable technologies like EXFEM has to be incorporated in industrial scale to lessen the production cost. The current study may pave the way for the utilization of this technology on an industrial scale for various products.

### Supplementary Information

Supplementary Information.

## Data Availability

All data generated or analyzed during this study are included in this published article [and its supplementary information files]. For further information on any data, please contact the corresponding author Dr. Senthilkumar Rathnasamy (senthilrathna@sastra.ac.in).
