# Supplementary material for: Sustainable production and preparative purification of thermostable alkaline α-amylase by Bacillus simplex (ON754233) employing natural deep eutectic solvent-based extractive fermentation
Source: Sci Rep. 2024 Jan 4;14:481. doi: 10.1038/s41598-024-51168-7 (PMC10766970; doi:10.1038/s41598-024-51168-7)
Supplement: Supplementary file 1 — Supplementary Information. [file 41598_2024_51168_MOESM1_ESM.docx]

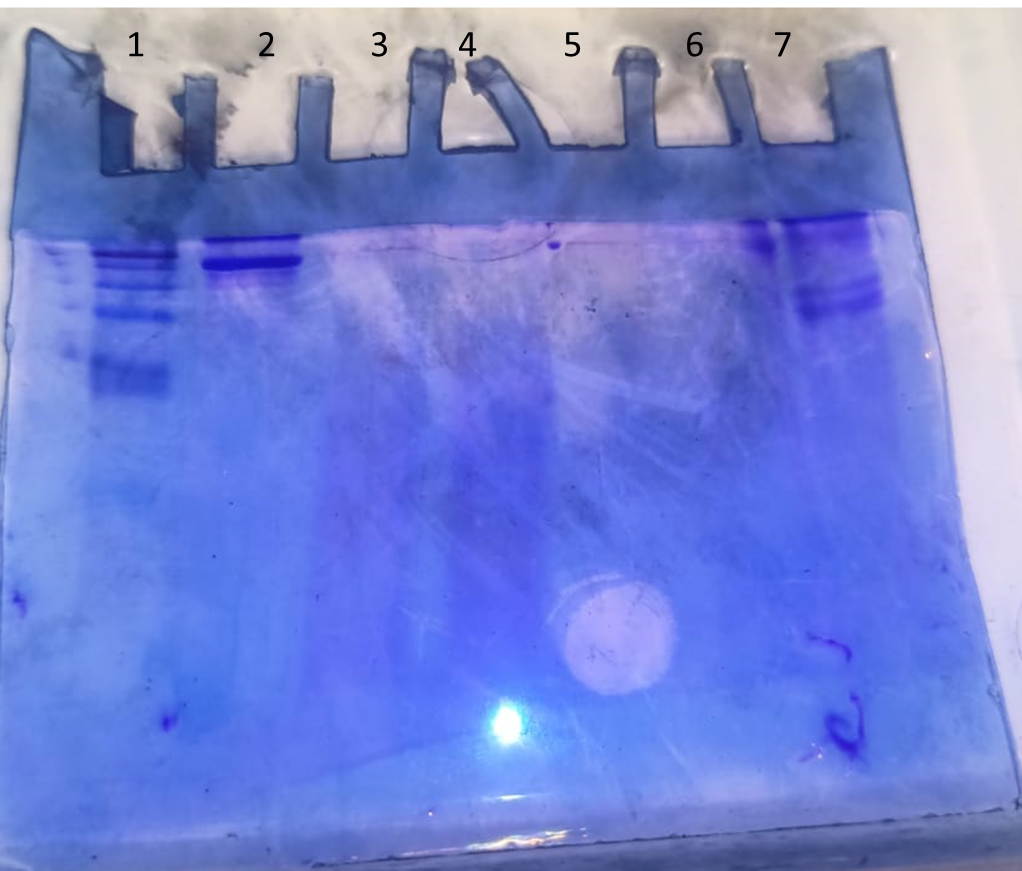


**S1- SDS PAGE electrophoresis.**

The SDS-PAGE electrophoresis was run for the partially recovered sample from extractive fermentation and purified sample from Gel filtration chromatography. Lane 1- High molecular weight marker (180 to 10 kDa), Lane 2 – GFC elute with a clear single band, Lane 3,4,5,6- No samples, Lane 7- Extractive fermentation crude along with other impurities
